# Missouri Valley Veterinary Association

**Published:** 1895-01

**Authors:** S. L. Hunter

**Affiliations:** Secretary; Fort Leavenworth, Kansas


Secretary.

### THE MISSOURI VALLEY VETERINARY ASSOCIATION.

THE regular meeting of the Missouri Valley Veterinary Association was held Wednesday evening, December 12, 1894, at the Byram Hotel, in Atchison, Kansas, and for the members who were present a rare treat certainly awaited them.

President Stewart was there with his stereopticon, and, assisted by Dr. Wattles, the society was most profitably instructed in the minor details of "Meat Inspection;" President Stewart explaining each plate exposed in such a lucid manner that none could fail to understand hog-cholera, swine-plague, tuberculosis, pleuro-pneumonia, as well as a great many physiological and pathological specimens; and one and all were of incalculable benefit to the veterinarian.

Many communications were read. The society unanimously decided to use its influence to induce the United States Veterinary Medical Association to hold its annual meeting at Des Moines, Iowa, in 1895.

*Resolved*, That the Missouri Valley Veterinary Association heartily indorse the desire of the Iowa veterinarians, that the next annual meeting of the United States Veterinary Medical Association be held at Des Moines, Ia.

R. H. HARRISON, Atchison,
J. MCCURDY, Topeka.
Committee.

The resolution was carried.

Dr. Wattles' report as delegate to the United States Veterinary Medical Association was presented and ordered filed. A vote of thanks was given to Dr. Wattles for the interesting and valuable report.

Dr. McCurdy's well-prepared paper on "Veterinary Legislation" elicited much discussion, resulting in the Chair appointing Drs. McCurdy, Mayo, and Hunter a committee on legislation.

Dr. S. L. Hunter read a paper on "Tuberculosis" and "Tuberculin." The most original paper was Dr. Wattles' "Treatment of Ossific Diseases of Articulations of the Horse by Hypodermatic Injections of Iodine." String halt and eserine claimed their share of discussion.

Adjourned to meet at Topeka, the second Wednesday of February, 1895.

This meeting was a record-breaker in regard to its enthusiasm and unqualified success.

Very truly,

S. L. HUNTER, V.S.,
Secretary.

FORT LEAVENWORTH, KANSAS, December 19, 1894.

